# Obstetric Interventions Among Native and Migrant Women: The (Over)use of Episiotomy in Portugal

**DOI:** 10.3389/ijph.2024.1606296

**Published:** 2024-03-21

**Authors:** Elsa Lorthe, Milton Severo, Sousan Hamwi, Teresa Rodrigues, Cristina Teixeira, Henrique Barros

**Affiliations:** ^1^ EPIUnit - Instituto de Saúde Pública, Universidade do Porto, Porto, Portugal; ^2^ Laboratório para a Investigação Integrativa e Translacional em Saúde Populacional (ITR), Porto, Portugal; ^3^ Unit of Population Epidemiology, Department of Primary Care Medicine, Geneva University Hospitals, Geneva, Switzerland; ^4^ Departamento de Ciências de Saúde Pública e Forenses e Educação Médica, Faculdade de Medicina da Universidade do Porto, Porto, Portugal; ^5^ Department of Gynecology and Obstetrics, Centro Hospitalar Universitário São João, Porto, Portugal; ^6^ Polytechnic Institute of Bragança, Bragança, Portugal

**Keywords:** episiotomy, migrant, differential care, obstetric interventions, healthcare inequities, perinatal health, reproductive health

## Abstract

**Objective:** Episiotomy, defined as the incision of the perineum to enlarge the vaginal opening during childbirth, is one of the most commonly performed surgical interventions in the world. We aimed to determine if migrant status is associated with episiotomy, and if individual characteristics mediate this association.

**Methods:** We analyzed data from the Bambino study, a national, prospective cohort of migrant and native women giving birth at a public hospital in mainland Portugal between 2017 and 2019. We included all women with vaginal delivery. The association between migrant status and episiotomy was assessed using multivariable multilevel random-effect logistic regression models. We used path analysis to quantify the direct, indirect and total effects of migrant status on episiotomy.

**Results:** Among 3,583 women with spontaneous delivery, migrant parturients had decreased odds of episiotomy, especially those born in Africa, compared to native Portuguese women. Conversely, with instrumental delivery, migrant women had higher odds of episiotomy. Disparities in episiotomy were largely explained by maternity units’ factors, and little by maternal and fetal characteristics.

**Conclusion:** Our results suggest non-medically justified differential episiotomy use during childbirth and highlight the importance of developing evidence-based recommendations for episiotomy use in a country with a high frequency of medical interventions during delivery.

## Introduction

The population of European countries comprises a growing share of international migrants, amounting to almost 10% in 2017 [1]. Migration is a core determinant of health and wellbeing [2], and encompasses a range of public health challenges and opportunities that have broadened over time [3]. This has been accompanied by a paradigm shift in the discourse on migration and health, from a focus on national borders and health security to issues of equity, right to health, social determinants of health and universal health coverage [1]. In the last years, the World Health Organisation has given priority to promoting the health status of migrants through different action plans and resolutions [1]. Migrant health has also been included in a number of Sustainable Development Goals (SDGs) and its central principle of “leaving no one behind,” because of its role as both a catalyst and a driver of sustainable development [3].

Addressing the needs of migrants across a wide range of health dimensions (such as non-communicable diseases, occupational health, and maternal and child health) is not only a matter of social justice but also a crucial issue for global health and wellbeing [1, 3]. Improving migrant maternal health has emerged as a top priority for many stakeholders in high-income countries, due to the large influx of migrant women of reproductive age (in Europe, 21% of babies are born to foreign-born women [4, 5]), the importance of adequate maternal care in the health of pregnant women and their offspring, and previously observed migrant-native disparities in perinatal health [1, 6–9]. Migrant women may have better perinatal health than native women, explained by the healthy migrant effect, or conversely be more vulnerable to maternal and child health problems, due to socioeconomic circumstances, health background and the existence of health inequalities or language barriers [10–17]. Studies comparing migrants with natives often focus on assessing health outcomes. However, the evaluation of medical practices is relevant as well, as some are considered as measurable indicators of the quality of obstetric care. A better comprehension of obstetric care provision is important to enhance the equity of service provision, improve overall maternal and neonatal health and support evidence-informed perinatal health policy-making [18].

Episiotomy is the incision of the perineum to enlarge the vaginal opening during the second stage of childbirth. The first documented episiotomy dates back to 1741 [19]. In the first half of the 20th century, more and more women were giving birth in hospital and doctors were managing normal uncomplicated deliveries, leading to a considerable increase in episiotomy rates. Historically, it was used to facilitate the second stage of labor, prevent maternal and neonatal trauma associated with delivery, and reduce long-term complications [20]. Since then, episiotomy has become one of the most commonly performed surgical interventions in the world [20, 21], ranging from less than 10% of all deliveries in Scandinavian countries to 100% in Taiwan [22, 23]. In Portugal, episiotomy rates remain high, reaching 72.9% in 2010 (66.9% with non-instrumental deliveries and 94.4% with instrumental deliveries) [22]. A significant decrease over time was observed in spontaneous deliveries (from 81.5% in 2000 to 54.0% in 2015) but not in instrumental deliveries (95.5% in 2000, 94.0% in 2015) [24].

Commonly reported indications for episiotomy include fetal distress, potentially complicated births (e.g., breech or shoulder dystocia), forceps or vacuum deliveries (in order to prevent obstetric anal sphincter injury), large babies, and preterm births [25–28]. However, these indications are based on tradition or clinical experience [29], with limited scientific evidence to support them [21, 27], whereas the contribution of episiotomy to increased maternal morbidity is well established [20, 26]. As a result, most clinical guidelines recommend against the routine use of episiotomy, but remain vague about specific indications [26, 30], or refer only to situations in which episiotomy is not recommended [21, 28–34]. Uncertainty remains regarding the benefits of performing an episiotomy with instrumental delivery [20, 27, 28]. Alarmingly, a significant number of episiotomies are performed without any clinical indication [35].

These differences in policies and practices, reflected by large variations across and within countries, suggest that the decision to perform an episiotomy can be influenced by the practitioner’s personal knowledge, attitude, profession (midwife or clinical doctor), experience, and local culture [35–37]. Parturients’ characteristics also affect the use of episiotomy which varies according to socioeconomic characteristics [37], such as ethnicity [38–40] or insurance [39]. However, it is unclear whether migrant status is an independent risk factor or is associated with episiotomy through maternal or fetal characteristics, or medical practices.

We aimed to determine if migrant status is associated with episiotomy, and if individual characteristics mediate this association.

## Methods

### Setting and Data Collection

We used baseline data from the Bambino study, a national, prospective, observational cohort study that aimed to investigate the maternity experiences of migrant and native women giving birth in mainland Portugal [15]. All 39 Portuguese public maternity units (accounting for 85% of all deliveries in mainland Portugal in 2018) were invited to participate in the project, and 32 (82%) agreed to collaborate. Between April 2017 and March 2019, the clinical staff on duty in the collaborating maternity units invited adult (aged 18+) migrant (i.e., foreign-born) women who delivered a live-born baby to participate during their hospital stay for delivery. For each migrant woman recruited, a native Portuguese woman who had the following live birth at the same hospital was also invited to participate. In total, 5,431 women gave their written consent and were included.

### Ethics

The Bambino study was approved by the Ethics Commission of the Institute of Public Health of the University of Porto (CE14013, 14 March 2014), by the local Ethics Committees of all the participating hospitals and by the National Commission for Data Protection (Authorization No. 13585/2016, 28 December 2016). Patients were not involved in defining the research questions or designing the study.

### Migration and Maternity Care in Portugal

International migrants represented 8.5% of the Portuguese population in 2018 [1], with a large share of women of reproductive age [41–43]. Almost half of the foreign-born residents in Portugal come from Brazil and Portuguese speaking African countries, due to long term and settled migration linked to former colonial ties up to the 1970s [42]. The Portuguese National Health Service guarantees universal free access to maternity care services for all pregnant women, regardless of their country of origin, nationality or legal status [44]. However, Portugal’s integration policies in 2019 were above average in all policy areas compared to all other developed countries except migrant health, which was identified as the weakest area [45]. To our knowledge, there are no national guidelines for episiotomy, reflecting a more general lack of standards, clinical guidelines and quality improvement culture in Portugal [46].

### Study Population

**FIGURE 1:**
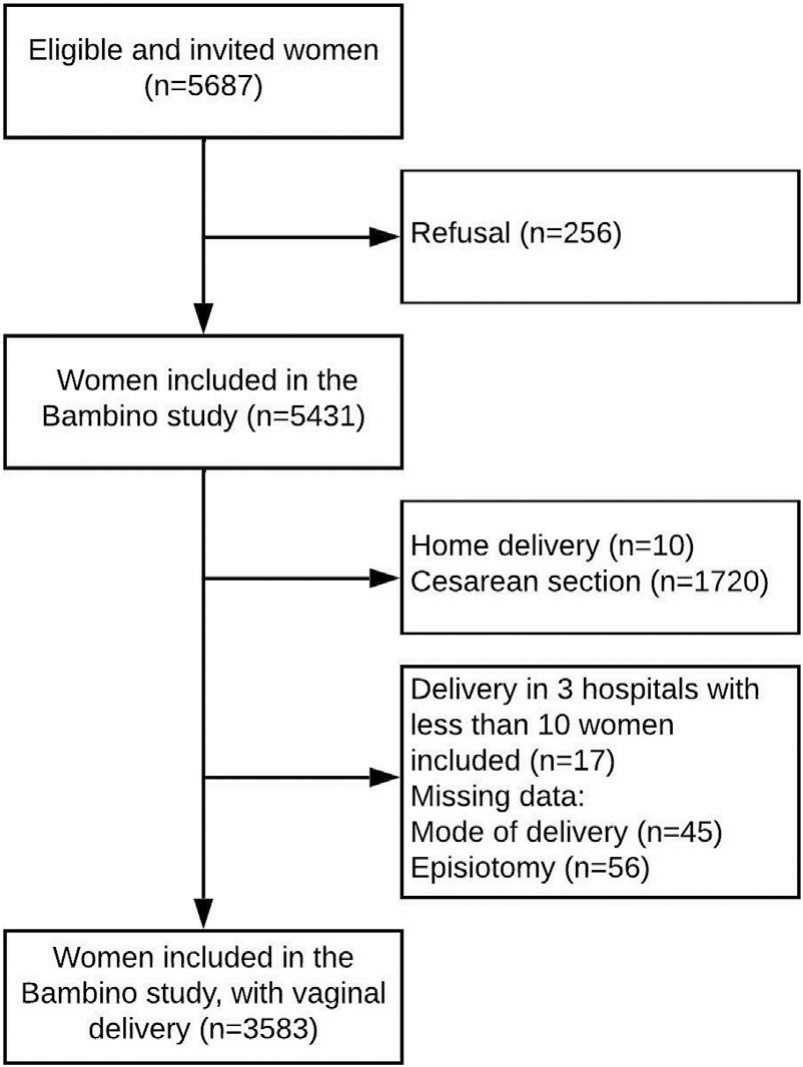
Flowchart of the study participants (Portugal, 2017–2019).

Our study population included all women with a vaginal delivery who delivered at one of the participating hospitals (Figure 1). We further excluded women with missing data on the mode of delivery (*n* = 45) and episiotomy (*n* = 56), as well as 17 women who delivered in three hospitals with very few participants in the study (in order to improve the convergence of our models).

### Main Exposure and Outcome Measures

The main exposure was migrant status, defined by the mother’s country of birth, as recommended by the “Reproductive Outcomes And Migration” (ROAM) collaboration and Euro-Peristat [4, 47]. Women were first classified as migrant (foreign-born) vs. native (Portuguese-born). Then, migrant women were grouped by geographical regions, classified according to the United Nations list of world macro-regions (Africa, America, Europe, Asia, Oceania). The outcome was episiotomy, a binary variable (yes vs. no), abstracted from medical records.

### Other Studied Factors

The following variables were included in the analysis: sociodemographic characteristics (maternal age, education, marital status), obstetric characteristics (parity, previous cesarean section, complications during pregnancy, multiple pregnancy, onset of labour, fetal presentation at delivery, mode of delivery [spontaneous vaginal delivery vs. instrumental vaginal delivery, i.e., the use of forceps or a ventouse suction cup to aid delivery of the fetus], severe perineal tears [i.e., grades 3–4 perineal tears], maternity unit, region of delivery) and neonatal characteristics (gestational age at birth, birthweight, fetal distress during expulsive efforts defined by Apgar score <7 at 1 min as a proxy).

### Statistical Analysis

Maternal and neonatal characteristics and the outcome were described as frequencies and percentages. Binomial confidence intervals were calculated for episiotomy proportions. As confounder assessment in exposed and non-exposed participants should not be based on *p*-values, we did not present inferential statistics [48]. We investigated interactions between migrant status and relevant covariates (including education and parity), and found a strong interaction with the mode of delivery (spontaneous vs. instrumental, *p* < 0.001). An interaction term (migrant status*mode of delivery) was therefore included in all models.

The association between migrant status and episiotomy was assessed using univariable and multivariable multilevel random-effect (with maternity units as level 2) logistic regression models and quantified by odds ratios (OR) and 95% confidence intervals (95% CI). Multivariable logistic regression models were adjusted for confounding (sociodemographic characteristics, i.e., maternal age and maternal education, Model 1) and intermediate factors (maternal and neonatal risk factors for episiotomy, namely, parity, multiple pregnancy, induced labour, fetal presentation, gestational age, birthweight and Apgar at 1 min < 7 as a proxy for fetal distress during expulsion, Model 2). A sensitivity analysis was carried out by restricting the sample to women with a low-risk pregnancy, defined as parturients with a singleton pregnancy, cephalic presentation, delivering at term (≥37 weeks) a baby of normal weight (2,500–3,999 grams). The proportion of missing data ranged from 0% to 5.4% for each covariate. Multiple imputation by chained equations involved using all baseline variables and outcome, with a logistic regression imputation model for binary variables and a multinomial imputation model for categorical variables. We generated 50 imputed datasets with 20 iterations, and results were pooled according to Rubin’s rules (Model 3).

We used path analysis to quantify the direct, indirect (through the relation with mediators, namely, parity, birthweight and low Apgar score, which, in turn, were significantly associated with the odds of episiotomy in the logistic regression model) and total effect (the sum of the coefficients of direct and indirect effects) of migrant status on episiotomy. This method permits to decompose and compare the magnitudes of effects between variables with complex inter-relations or test mediation effects. We assumed a causal/temporal relationship between migrant status and episiotomy, taking into account a relevant set of potential confounders and mediator-outcome confounders (Supplementary Figure S1). Regression coefficients were obtained via adjusted logistic regression models carried out on complete observations and fitted with Mplus software version 6.12 (Muthén and Muthén, Los Angeles, California); 95% CIs were calculated by bootstrapping with 1,000 replications. Goodness of fit was evaluated using the Confirmatory Fit Index (CFI; good fit ≥0.95; acceptable fit ≥0.90) and Root Mean Square Error of Approximation (RMSEA; good fit: <0.06; acceptable fit <0.08).

Finally, we used median odds ratios (MOR) to quantify the magnitude of variability in the use of episiotomy among maternity units (second-level variation). The MOR corresponds to the median value of the OR obtained when comparing the odds of having episiotomy among two randomly selected women (with identical covariates) from two randomly chosen units, when units are ordered by risk [49]. In other words, the MOR indicates the extent to which a woman’s probability of episiotomy is determined by the maternity unit; it is comparable to an OR used for patient-level factors. MORs were calculated using multilevel logistic regressions, and their 95% CI were estimated by bootstrapping. The first model was adjusted for migrant status, mode of delivery and interaction between these two variables, the second one for known individual risk factors, i.e., those clinically relevant or found in the literature (maternal age, education, parity, multiple pregnancy, induced labour, mode of delivery, mode of delivery*migrant status, fetal presentation, gestational age, birthweight and Apgar at 1 min < 7 as a proxy for fetal distress during expulsion). An MOR is equal to 1.0 (meaning no differences between maternity units in the odds of a woman undergoing an episiotomy) or higher; a greater variation between units results in a larger MOR. Stata/IC 16 was used for data analysis. Statistical significance was set at two-tailed *p* < 0.05.

## Results

A total of 3,583 women from 29 maternity units met the inclusion criteria (Figure 1). Of them, 1,722 were born in Portugal, and 1,861 were migrants. Among migrant women, 51.8% were born in Africa, 20.3% in America, 21.7% in Europe and 6.2% in Asia. Sociodemographic, obstetric and neonatal characteristics are presented in Table 1. Compared to native women, migrant parturients were more often multiparous and had a higher frequency of spontaneous delivery. Their babies displayed higher birthweights and were more likely to have an Apgar score below 7 at 1 min.

**TABLE 1:** Sociodemographic, obstetric and neonatal characteristics by migrant status, overall and by region of origin (Portugal, 2017–2019).

	Native women	Migrant women				
	Native women (*n* = 1,722) n (%)	All migrant women (*n* = 1,861) n (%)	Africa (*n* = 963) n (%)	America (*n* = 378) n (%)	Europe (*n* = 404) n (%)	Asia (*n* = 116) n (%)
Sociodemographic characteristics						
Maternal age (years) (*n* = 3,572)						
<20	109 (6.3)	83 (4.5)	47 (4.9)	13 (3.5)	19 (4.7)	4 (3.5)
21–34	1,179 (68.6)	1,362 (73.5)	726 (75.7)	255 (67.6)	286 (71.0)	95 (82.6)
≥35	430 (25.1)	409 (22.0)	186 (19.4)	109 (28.9)	98 (24.3)	16 (13.9)
Maternal education (*n* = 3,439)						
Master or PhD	129 (7.7)	96 (5.4)	21 (2.3)	24 (6.7)	37 (9.8)	14 (12.8)
Bachelor	402 (24.1)	437 (24.7)	201 (21.8)	75 (20.8)	129 (34.3)	32 (29.4)
Secondary school (12 years)	541 (32.4)	674 (38.1)	336 (36.4)	185 (51.4)	119 (31.7)	34 (31.2)
Basic (9 years)	437 (26.1)	356 (20.2)	221 (23.9)	56 (15.6)	60 (16.0)	19 (17.4)
Primary (4 years) or None	162 (9.7)	205 (11.6)	144 (15.6)	20 (5.5)	31 (8.2)	10 (9.2)
Married or living with a partner (*n* = 3,564)	1,204 (70.1)	1,291 (69.9)	569 (59.5)	284 (75.7)	338 (84.7)	100 (86.2)
Region of delivery (*n* = 3,583)						
North	386 (22.4)	311 (16.7)	62 (6.4)	104 (27.5)	115 (28.5)	30 (25.9)
Center	168 (9.8)	163 (8.8)	25 (2.6)	53 (14.0)	69 (17.1)	16 (13.8)
Lisbon and Tagus Valley	1,100 (63.9)	1,267 (68.1)	864 (89.7)	194 (51.3)	143 (35.4)	66 (56.9)
South	68 (3.9)	120 (6.4)	12 (1.3)	27 (7.2)	77 (19.0)	4 (3.4)
Obstetric characteristics						
Parity (*n* = 3,428)						
Primiparous	803 (48.4)	785 (44.3)	368 (40.7)	179 (49.3)	184 (47.3)	54 (47.4)
Multiparous, no previous cesarean	730 (44.0)	821 (46.4)	441 (48.8)	145 (39.9)	182 (46.8)	53 (46.5)
Multiparous, with previous cesarean	125 (7.6)	164 (9.3)	95 (10.5)	39 (10.8)	23 (5.9)	7 (6.1)
Multiple pregnancy (*n* = 3,583)	20 (1.2)	16 (0.9)	11 (1.1)	3 (0.8)	2 (0.5)	0 (0.0)
Any complications during pregnancy (*n* = 3,501)	440 (26.0)	488 (27.0)	250 (26.8)	91 (24.8)	107 (27.0)	40 (35.4)
Cephalic presentation (*n* = 3,528)	1,682 (99.6)	1,831 (99.6)	956 (100)	368 (99.5)	392 (98.5)	115 (100)
Onset of labour (*n* = 3,473)						
Spontaneous	1,248 (75.4)	1,339 (73.6)	690 (73.1)	275 (75.1)	296 (74.6)	78 (70.3)
Induced	407 (24.6)	479 (26.4)	254 (26.9)	91 (24.9)	101 (25.4)	33 (29.7)
Mode of delivery (*n* = 3,583)						
Spontaneous	1,333 (77.4)	1,505 (80.9)	825 (85.7)	288 (76.2)	316 (78.2)	76 (65.5)
Instrumental	389 (22.6)	356 (19.1)	138 (14.3)	90 (23.8)	88 (21.8)	40 (34.5)
Episiotomy (*n* = 3,583)	905 (52.6)	894 (48.0)	377 (39.2)	211 (55.8)	233 (57.7)	73 (62.9)
Episiotomy among women with spontaneous delivery (*n* = 2,838)	587/1,333 (44.0)	572/1,505 (38.0)	256/825 (31.0)	128/288 (44.4)	152/316 (48.1)	36/76 (47.4)
Episiotomy among women with instrumental delivery (*n* = 745)	318/389 (81.8)	322/356 (90.5)	121/138 (87.7)	83/90 (92.2)	81/88 (92.1)	37/40 (92.5)
Severe perineal tears (*n* = 3,388)	8 (0.5)	16 (0.9)	6 (0.7)	4 (1.1)	2 (0.5)	4 (3.6)
Neonatal characteristics						
Gestational age (weeks) (*n* = 3,522)						
<37	119 (7.0)	99 (5.4)	50 (5.3)	23 (6.2)	19 (4.8)	7 (6.0)
37	122 (7.2)	139 (7.6)	75 (7.9)	30 (8.2)	27 (6.8)	7 (6.0)
38	295 (17.4)	274 (15.0)	147 (15.5)	51 (13.8)	63 (15.8)	13 (11.2)
39	521 (30.8)	562 (30.7)	271 (28.7)	123 (33.3)	128 (32.1)	40 (34.5)
40	467 (27.6)	529 (28.9)	287 (30.3)	97 (26.3)	113 (28.4)	32 (27.6)
≥41	169 (10.0)	226 (12.4)	116 (12.3)	45 (12.2)	48 (12.1)	17 (14.7)
Birthweight (grams) (*n* = 3,583)						
<2,500	120 (7.0)	89 (4.8)	45 (4.7)	17 (4.5)	20 (5.0)	7 (6.0)
2,500–2,999	449 (26.1)	405 (21.8)	235 (24.4)	76 (20.1)	70 (17.3)	24 (20.7)
3,000–3,499	715 (41.5)	831 (44.6)	417 (43.3)	166 (43.9)	192 (47.5)	56 (48.3)
3,500–3,999	373 (21.6)	432 (23.2)	207 (21.5)	99 (26.2)	102 (25.2)	24 (20.7)
≥4,000	65 (3.8)	104 (5.6)	59 (6.1)	20 (5.3)	20 (5.0)	5 (4.3)
Apgar <7 at 1 min (*n* = 3,458)	38 (2.3)	67 (3.7)	39 (4.2)	14 (3.9)	9 (2.3)	5 (4.4)

The overall frequency of episiotomy was 50.2% (95% CI 48.6–51.9). Differences in the frequency of episiotomy were observed by migrant status (52.6% [95% CI 50.2–54.9] in native women vs. 48.0% [45.7–50.3] in migrant women) and by mode of delivery (40.8% [39.0–42.7] with spontaneous vaginal delivery vs. 85.9% [83.2–88.3] with instrumental delivery). Among migrant women, those born in Africa had a lower frequency of episiotomy (39.2% [36.1–42.3]) than those born in other regions. Similar trends were observed after stratification by mode of delivery (Table 1). Other characteristics associated with increased use of episiotomy were: being primiparous, having a large baby and having a newborn with a low Apgar score (Table 2). Among women with spontaneous delivery, multivariable analyses showed decreased odds of episiotomy in migrant women (aOR 0.72 [0.60–0.85]), especially in those born in Africa (aOR 0.57 [0.46–0.71]) compared to Portuguese women. Conversely, among women with instrumental delivery, migrant women had higher odds of episiotomy (aOR 2.62 [1.32–5.20]) compared to native women, but no significant difference was observed when stratifying by region of birth (Table 3). Similar results were shown among women with a low-risk pregnancy (Table 4).

**TABLE 2:** Sociodemographic, obstetric and neonatal characteristics of women delivering vaginally with and without episiotomy, stratified by the mode of delivery (Portugal, 2017–2019).

	Spontaneous delivery (*n* = 2,838)		Instrumental delivery (*n* = 745)	
	No episiotomy (*n* = 1,679) n (%)	Episiotomy (*n* = 1,159) n (%)	No episiotomy (*n* = 105) n (%)	Episiotomy (*n* = 640) n (%)
Sociodemographic characteristics				
Maternal age (years) (*n* = 3,572)				
<20	68 (4.1)	81 (7.0)	3 (2.9)	40 (6.3)
21–34	1,160 (69.2)	853 (73.9)	65 (61.9)	463 (72.7)
≥35	447 (26.7)	221 (19.1)	37 (35.2)	134 (21.0)
Maternal education (*n* = 3,439)				
Master or PhD	73 (4.5)	81 (7.3)	10 (9.7)	61 (10.0)
Licence	347 (21.4)	292 (26.4)	27 (26.2)	173 (28.3)
Secondary school	514 (31.8)	432 (39.1)	34 (33.0)	235 (38.4)
Basic (9 years)	441 (27.2)	220 (19.9)	24 (23.3)	108 (17.6)
Primary (4 years) or None	244 (15.1)	80 (7.3)	8 (7.8)	35 (5.7)
Married or living with a partner (*n* = 3,564)	1,170 (70.0)	788 (68.2)	82 (78.1)	455 (71.9)
Obstetric characteristics				
Parity (*n* = 3,428)				
Primiparous	399 (24.9)	680 (61.9)	48 (47.1)	461 (73.9)
Multiparous, no previous cesarean	1,083 (67.5)	333 (30.3)	39 (38.2)	96 (15.4)
Multiparous, with previous cesarean	122 (7.6)	85 (7.8)	15 (14.7)	67 (10.7)
Multiple pregnancy (*n* = 3,583)	15 (0.9)	16 (1.4)	0 (0)	5 (0.8)
Any complications during pregnancy (*n* = 3,501)	454 (27.6)	279 (24.8)	31 (29.8)	164 (26.2)
Cephalic presentation (*n* = 3,528)	1,643 (99.6)	1,131 (99.5)	103 (100)	636 (99.7)
Onset of labour (*n* = 3,473)				
Spontaneous	1,245 (76.4)	848 (75.3)	66 (65.4)	428 (69.5)
Induced	385 (23.6)	278 (24.7)	35 (34.6)	188 (30.5)
Severe perineal tears (*n* = 3,388)	7 (0.4)	4 (0.4)	2 (2.1)	11 (1.8)
Neonatal characteristics				
Gestational age (weeks) (*n* = 3,522)				
<37	103 (6.2)	91 (8.0)	3 (2.9)	21 (3.3)
37	117 (7.1)	85 (7.5)	11 (10.6)	48 (7.6)
38	296 (17.9)	166 (14.6)	20 (19.2)	87 (13.8)
39	532 (32.2)	355 (31.3)	25 (24.0)	171 (27.2)
40	438 (26.5)	313 (27.6)	30 (28.9)	215 (34.1)
≥41	167 (10.1)	125 (11.0)	15 (14.4)	88 (14.0)
Birthweight (grams) (*n* = 3,583)				
<2,500	92 (5.5)	86 (7.4)	4 (3.8)	27 (4.2)
2,500–2,999	434 (25.9)	266 (23.0)	34 (32.4)	120 (18.8)
3,000–3,499	727 (43.3)	475 (41.0)	43 (40.9)	301 (47.0)
3,500–3,999	355 (21.1)	273 (23.5)	23 (21.9)	154 (24.1)
≥4,000	71 (4.2)	59 (5.1)	1 (1.0)	38 (5.9)
Apgar < 7 at 1 min (*n* = 3,458)	22 (1.4)	41 (3.7)	4 (4.1)	38 (6.2)

**TABLE 3:** Association of migrant status and episiotomy among women with spontaneous and instrumental delivery (Portugal, 2017–2019).

		Episiotomy	No episiotomy	Univariable analysis^a^ (*n* = 3,583)	Model 1—Multivariable analysis^b^, adjustment for confounders only (*n* = 3,431)	Model 2—multivariable analysis^c^, complete-cases analysis (*n* = 3,037)	Model 3—multivariable analysis^c^, multiple imputations (*n* = 3,583)
		n (%)	n (%)	OR (95% CI)	aOR (95% CI)	aOR (95% CI)	aOR (95% CI)
Spontaneous delivery (*n* = 2,838)	Migrant status	(*n* = 1,159)	(*n* = 1,679)				
	Native	587 (50.7)	746 (44.4)	Ref	Ref	Ref	Ref
	Migrant	572 (49.3)	933 (55.6)	**0.78 (0.67–0.91)**	**0.78 (0.66–0.92)**	**0.74 (0.61–0.90)**	**0.72 (0.60–0.85)**
	Region of birth						
	Portugal	587 (50.7)	746 (44.4)	Ref	Ref	Ref	Ref
	Africa	256 (22.1)	569 (33.9)	**0.63 (0.52–0.76)**	**0.64 (0.52–0.78)**	**0.58 (0.46–0.73)**	**0.57 (0.46–0.71)**
	America	128 (11.0)	160 (9.5)	1.01 (0.77–1.32)	0.98 (0.74–1.31)	0.90 (0.64–1.25)	0.85 (0.63–1.16)
	Europe	152 (13.1)	164 (9.8)	0.99 (0.76–1.30)	0.98 (0.73–1.30)	1.01 (0.73–1.41)	0.97 (0.72–1.30)
	Asia	36 (3.1)	40 (2.4)	1.08 (0.67–1.76)	1.00 (0.60–1.67)	1.32 (0.73–2.40)	1.13 (0.66–1.94)
Instrumental delivery (*n* = 745)	Migrant status	(*n* = 640)	(*n* = 105)				
	Native	318 (49.7)	71 (67.6)	Ref	Ref	Ref	Ref
	Migrant	322 (50.3)	34 (32.4)	**2.05 (1.09–3.85)**	**2.16 (1.12–4.16)**	**2.63 (1.24–5.57)**	**2.62 (1.32–5.20)**
	Region of birth						
	Portugal	318 (49.7)	71 (67.6)	Ref	Ref	Ref	Ref
	Africa	121 (18.9)	17 (16.2)	1.69 (0.76–3.77)	1.69 (0.73–3.91)	1.97 (0.75–5.18)	1.97 (0.83–4.72)
	America	83 (13.0)	7 (6.7)	2.36 (0.75–7.42)	2.51 (0.77–8.12)	3.29 (0.83–12.96)	3.24 (0.92–11.40)
	Europe	81 (12.6)	7 (6.7)	2.34 (0.75–7.32)	2.74 (0.81–9.29)	3.32 (0.88–12.61)	3.15 (0.93–10.74)
	Asia	37 (5.8)	3 (2.8)	2.76 (0.46–16.66)	2.65 (0.42–16.87)	3.27 (0.43–25.15)	3.88 (0.55–27.18)

^a^ Multilevel logistic regression model with an interaction term (migrant status*mode of delivery or region of birth*mode of delivery depending on the exposure considered).

^b^ Multilevel logistic regression model with an interaction term (migrant status*mode of delivery or region of birth*mode of delivery depending on the exposure considered), adjusted for maternal age and maternal education.

^c^ Multilevel logistic regression model with an interaction term (migrant status*mode of delivery or region of birth*mode of delivery depending on the exposure considered), adjusted for maternal age, maternal education, parity, multiple pregnancy, induced labour, presentation, gestational age, birthweight, Apgar < 7 at 1 min.

Bold values indicate significant assocations.

**TABLE 4:** Association of migrant status and episiotomy among women with a low-risk pregnancy (Portugal, 2017–2019).

		Episiotomy	No episiotomy	Univariable analysis^a^ (*n* = 3,085)	Model 1—Multivariable analysis^b^, adjustment for confounders only (*n* = 2,955)	Model 2—multivariable analysis^c^, complete-cases analysis (*n* = 2,665)	Model 3—multivariable analysis^c^, multiples imputations (*n* = 3,085)
		n (%)	n (%)	OR (95% CI)	aOR (95% CI)	aOR (95% CI)	aOR (95% CI)
Spontaneous delivery (*n* = 2,419)	Migrant status	(*n* = 974)	(*n* = 1445)				
	Native	490 (50.3)	635 (43.9)	Ref	Ref	Ref	Ref
	Migrant	484 (49.7)	810 (56.1)	**0.78 (0.66–0.92)**	**0.78 (0.65–0.93)**	**0.77 (0.62–0.94)**	**0.72 (0.60–0.87)**
	Region of birth						
	Portugal	490 (50.3)	635 (43.9)	Ref	Ref	Ref	Ref
	Africa	211 (21.6)	495 (34.3)	**0.62 (0.50–0.76)**	**0.63 (0.51–0.79)**	**0.59 (0.46–0.76)**	**0.58 (0.46–0.74)**
	America	111 (11.4)	138 (9.6)	1.03 (0.77–1.39)	1.01 (0.74–1.38)	0.94 (0.66–1.34)	0.87 (0.63–1.21)
	Europe	133 (13.7)	142 (9.8)	1.01 (0.75–1.35)	0.99 (0.73–1.34)	1.05 (0.74–1.48)	0.94 (0.68–1.29)
	Asia	29 (3.0)	35 (2.4)	1.00 (0.59–1.71)	0.92 (0.52–1.61)	1.35 (0.70–2.59)	1.08 (0.60–1.96)
Instrumental delivery (*n* = 666)	Migrant status	(*n* = 566)	(*n* = 100)				
	Native	277 (48.9)	69 (69.0)	Ref	Ref	Ref	Ref
	Migrant	289 (51.1)	31 (31.0)	**2.29 (1.17–4.45)**	**2.38 (1.19–4.77)**	**2.89 (1.31–6.37)**	**2.83 (1.37–5.83)**
	Region of birth						
	Portugal	277 (48.9)	69 (69.0)	Ref	Ref	Ref	Ref
	Africa	108 (19.1)	15 (15.0)	1.92 (0.82–4.53)	1.88 (0.77–4.59)	2.17 (0.78–6.08)	2.14 (0.85–5.39)
	America	74 (13.1)	6 (6.0)	2.85 (0.83–9.76)	2.90 (0.82–10.28)	3.94 (0.90–17.22)	3.80 (0.99–14.59)
	Europe	73 (12.9)	7 (7.0)	2.37 (0.73–7.70)	2.83 (0.80–9.95)	3.27 (0.83–12.84)	3.06 (0.86–10.86)
	Asia	34 (6.0)	3 (3.0)	2.84 (0.44–18.39)	2.75 (0.40–18.92)	3.53 (0.42–29.62)	4.08 (0.54–31.02)

^a^ Multilevel logistic regression model with an interaction term (migrant status*mode of delivery or region of birth*mode of delivery depending on the exposure considered).

^b^ Multilevel logistic regression model with an interaction term (migrant status*mode of delivery or region of birth*mode of delivery depending on the exposure considered), adjusted for maternal age and maternal education.

^c^ Multilevel logistic regression model with an interaction term (migrant status*mode of delivery or region of birth*mode of delivery depending on the exposure considered), adjusted for maternal age, maternal education, parity, induced labour, gestational age, birthweight, Apgar < 7 at 1 min.

Bold values indicate significant assocations.

Table 5 summarizes the path-analysis estimates for the decomposition of the total effect of migrant status into its direct and indirect (mediated by parity, low Apgar score and birthweight) components. The overall fit of the models was acceptable. In women with spontaneous vaginal delivery, the path-analysis estimates showed a total effect of migrant status on episiotomy (β −0.148, 95% CI −0.242; −0.046), mostly due to a direct effect (β −0.208, 95% CI −0.326; −0.095). There was also a significant indirect effect through low Apgar score, and non-significant indirect effects through parity and birthweight, resulting in the absence of an overall indirect effect. In women with instrumental delivery, total (β 0.432, 95% CI 0.189; 0.696) and direct (β 0.481, 95% CI 0.209; 0.748) effects were also found, as well as indirect effects through parity and birthweight, which worked in opposite directions; this difference explains the absence of an overall indirect effect.

**TABLE 5:** Decomposition of the total effect of migrant status on episiotomy into a direct effect and indirect effect mediated through parity, low Apgar score and birthweight, by path-analysis (Portugal, 2017–2019).

	Model 1	Model 2
	Spontaneous delivery	Instrumental delivery
Migrant status to episiotomy	β (95% CI)	β (95% CI)
Total effect	**−0.148 (**−**0.242;** −**0.046)**	**0.432 (0.189; 0.696)**
Direct effect	**−0.208 (**−**0.326**; −**0.095)**	**0.481 (0.209**; **0.748)**
Indirect effect	0.060 (−0.017; 0.148)	−0.049 (−0.166; 0.080)
Indirect effect through parity	−0.020 (−0.057; 0.012)	**−0.092 (**−**0.179**; −**0.029)**
Indirect effect through low Apgar at 1 min	**0.077 (0.019**; **0.166)**	0.021 (−0.026; 0.167)
Indirect effect through birthweight	0.003 (−0.002; 0.011)	**0.022 (0.001**; **0.070)**
RMSEA	0.060	0.039
CFI	0.865	0.921

CFI, confirmatory fit index; RMSEA, root mean square error of approximation; 95% CI, 95% confidence intervals.

Models were adjusted for maternal education, multiple pregnancy, induced labour, fetal presentation and gestational age (maternal age was excluded to improve the model fit because of its strong association with parity).

Bold values indicate significant assocations.

Finally, the frequency of episiotomy ranged from 5.3% to 90.6% across maternity units. The degree of between-units’ variation was high for episiotomy (MOR 1.81, 95% CI 1.39–2.20), indicating that the median odds of episiotomy were 1.8-fold higher if the same woman delivered in a maternity unit with a higher vs. lower prevalence of episiotomy. Additional adjustment for known individual risk factors of episiotomy increased the MOR estimate (2.06, 95% CI 1.55–2.58), indicating a clear heterogeneity across maternity units.

## Discussion

### Main Findings

In the Portuguese Bambino cohort, the frequency of episiotomy was high, with large heterogeneity across maternity units. We found that with spontaneous delivery, migrant women, especially those from African countries, had decreased odds of having an episiotomy, compared to native Portuguese women, independently of known sociodemographic and obstetric risk factors. However, with instrumental delivery, migrant women had increased odds of episiotomy. Path analysis showed that migrant status and episiotomy were mainly associated through a direct effect, suggesting that obstetrical and neonatal characteristics had little influence on the risk of episiotomy (and often in opposite directions that compensated for each other).

### Interpretation

Despite a decreasing trend in use over time [22, 24], episiotomy remains a frequent intervention in Portugal compared to most European countries [22], for instrumental deliveries and, to a lesser extent, spontaneous deliveries. This routine practice may reflect individual and local enrooted clinical habits (particularly with the use of forceps) [40, 50, 51], and/or the belief that episiotomy prevents severe perineal tears and is therefore beneficial to the parturient. This is not justified by current evidence [20, 21], contributes to maternal morbidity [20, 26], and raises questions on adherence to and effective implementation of evidence-based obstetrical practices and care at individual, institutional and national levels [52].

We also found differential frequencies of episiotomy by migrant status, with opposite directions depending on the mode of delivery. The former finding is consistent with previous research. In a study comparing 59,245 foreign-born women with 149,737 Australian-born women, episiotomy rates were higher in Asian and Sub-Saharan African women than among Australian women, but lower in Oceanian, North African and Middle Eastern women [38]. Similarly, a study of 11,540 women originating from 141 countries showed an increased risk of both episiotomy and operative vaginal delivery in African women compared with Norwegian women in a low-risk maternity unit [53]. However, instrumental deliveries also varied by maternal region of birth and the mode of delivery was not taken into account in their analysis. A French population-based study performed in 2016 showed that almost 20% of 9,284 parturients had an episiotomy, with significant variations between maternity units that were not explained by individual characteristics [54]. The main risk factors identified in both primiparas and multiparas were maternal birth in Africa, macrosomia and instrumental delivery, in line with our results. A repeated survey on childbirth experiences of a nationally representative sample of women from the United States concluded that Medicaid users were significantly less likely to receive an episiotomy than women with another health insurance. In contrast, Medicaid users with stronger natural birth desires were more likely to receive one compared to non-Medicaid-recipients [55]. Our findings are consistent with this dual narrative in which minority women are receiving both less and more medical interventions during childbirth depending on other characteristics.

Our results go beyond previous studies by providing important insights into the mechanisms underlying these disparities. Using different methodological approaches, we suggest that, in Portugal, disparities in episiotomy among native and migrant parturients are largely explained by maternity units’ factors, and little by maternal and fetal characteristics, thereby reflecting non-medically justified differential care according to migrant status [56]. Several explanations can be evoked. The first one is related to the healthcare professional who attends the delivery. In Portugal, while midwives are usually responsible for low-risk spontaneous deliveries, obstetricians continue to play a leading role in spontaneous deliveries and their presence is required for instrumental and high-risk deliveries [57]. Obstetricians are known to perform episiotomies more often than midwives [40, 54]. However, it is difficult to disentangle the effect of the birth attendant itself from the delivery complications considered as indications for episiotomy. We did not have appropriate data on the healthcare professionals attending childbirth to test this hypothesis. Second, migrant status may affect how healthcare providers perceive patients’ ability to communicate and understand medical information [17, 55]. Migrant women might also have low health literacy and/or limited host-country language skills resulting in poorer empowerment and capacity to participate in medical decisions [58]. Communication difficulties prevent adequate information and consent [17, 18] and could lead to more obstetric interventions, particularly during instrumental delivery, which is a potentially stressful and painful emergency situation. Finally, healthcare professionals’ practices can be influenced by implicit biases, which refer to unconscious negative feelings or stereotypes against a social group or a person on the basis of irrelevant characteristics such as ethnicity or gender. Such biases may predispose caregivers in decision-making situations to treat people from different social groups differently, leading to lower quality of care [59, 60] and worst health outcomes [61]. Improving intercultural communication skills among healthcare providers and raising awareness of implicit bias [62] may help reduce the gap between the episiotomy rates of Portuguese and migrant women by preventing non-medically justified episiotomies.

### Strengths and Limitations

The main strengths of our study are its prospective and multicenter design, its large sample size and the diversity of countries of origin represented among participants. The inclusion of 29 maternity units allowed us to investigate a large variety of clinical practices. The use of path-analysis brought novel insights regarding the mechanisms underlying obstetric care inequities between migrants and natives and allowed us to identify intervention points.

Some limitations should be kept in mind. We had very little data on labour and delivery management, including the healthcare provider responsible for the birth, although some techniques are considered risk factors (oxytocin use) or protective factors (perineal massage, hot compresses, sitting-position and lateral position) [40]. However, the likelihood of a confounding bias is low as these variables cannot be considered as predictors of migrant status. We had no information on the indication for episiotomy, which are not always mentioned in medical reports and were not collected. However, we could take into account all the main indications reported in the literature, thereby ensuring the relevance of our results. We also had limited power to show differences in the frequency of episiotomy by region of birth after stratification. External validity is limited as each country has a unique history of migration flows (influenced by labor migration, historical ties between countries, established networks, etc.), however, health inequalities occur worldwide. To make our study comparable to the relevant literature, which is subject to the same limitations, we defined migrant status based on the most used indicator (country of birth) when investigating health outcomes of migrants [10]. The inclusion of private maternity units, where obstetric interventions are more frequent [40], would have impacted frequencies but probably not the associations found.

### Conclusion

These results suggest non-medically justified differential care during childbirth and highlight the importance of developing evidence-based recommendations for episiotomy use and implementing them into practices. This would improve the quality of obstetric care and benefit all women, whether they are migrants or natives. Reducing healthcare inequities and implementing more culturally sensitive maternity care policies will contribute to achieving the United Nations Sustainable Development Goals 2030.

## Supplementary Material

Supplementary Figure S1 Path analysis diagram for the effect of migrant status on episiotomy (Portugal, 2017–2019). The effect of migrant status on episiotomy is decomposed on direct and indirect effects through parity, fetal distress during expulsion and birthweight. Circled variables (Medical practices during delivery, Healthcare professional) are unobserved.
