# Supplementary figures and images for: Obstetric Interventions Among Native and Migrant Women: The (Over)use of Episiotomy in Portugal
Source: Int J Public Health. 2024 Mar 21;69:1606296. doi: 10.3389/ijph.2024.1606296 (PMC10991787; doi:10.3389/ijph.2024.1606296)

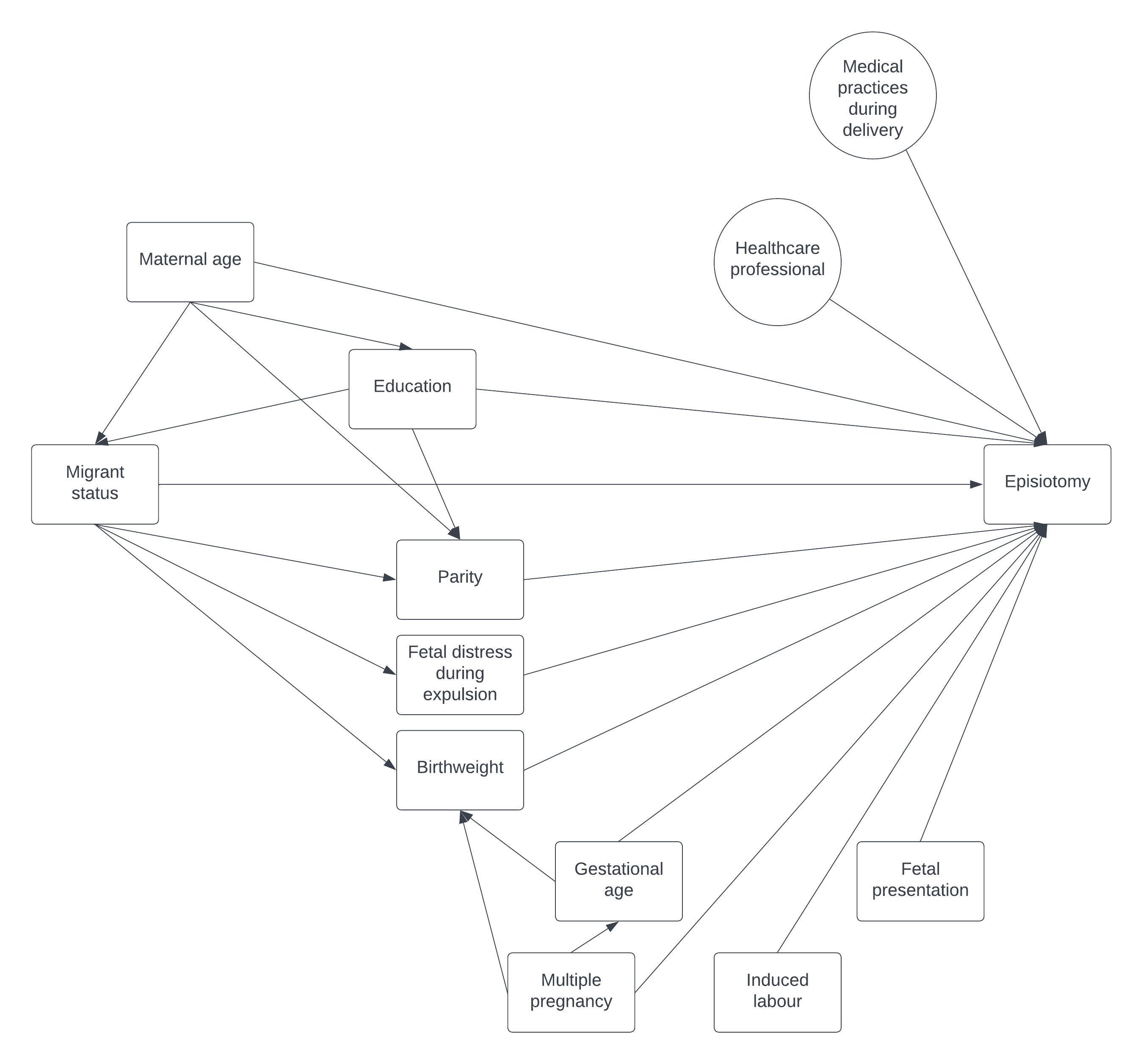

Supplement: Supplementary file 1 [file Image1.jpeg]
